# Transcriptome Analysis and Physiological Response to Salinity Stress in Adzuki Bean (*Vigna angularis*) at the Seedling Stage

**DOI:** 10.3390/plants14172722

**Published:** 2025-09-01

**Authors:** Baomei Wu, Ying Zhang, Qiang Zhang, Linlin Hao, Yanru Guo, Min Xu, Weizhong Liu, Binbin Wang

**Affiliations:** 1 School of Life Sciences, Shanxi Normal University, Taiyuan 030031, China; wubaomei@sxnu.edu.cn (B.W.); zy970411666@163.com (Y.Z.); zhangqiang@sxnu.edu.cn (Q.Z.); haol0102@163.com (L.H.); 17200640343@163.com (Y.G.); 2 School of Chemical Engineering and Technology, Tianjin University, Tianjin 300072, China; minxu@tju.edu.cn

**Keywords:** Adzuki bean, salinity stress, physicochemical properties, transcriptome profile

## Abstract

Adzuki bean (*Vigna angularis* (Willd.) Ohwi & H. Ohashi) is a significant crop for its applications in both traditional medicine and nutritional diets in China. However, there remains a paucity of exploration employing an RNA-seq approach to investigate the molecular response mechanisms of the species under salinity stress. In this study, Jin Xiao Dou 6 (JXD6) adzuki bean cultivar was subjected to 0 mmol/L (CK), 32.5 mmol/L, and 65.0 mmol/L NaCl treatments to preliminarily characterize salinity-induced alterations in plant height, chloroplast pigment contents, leaf surface humidity and temperature, H_2_O_2_ and O_2_^−^ accumulation, activities of antioxidative enzymes, and transcriptome profiles. Under increasing NaCl concentrations, the plant height of JXD6 seedlings was progressively inhibited. Conversely, the unifoliate leaves exhibited elevated leaf surface temperature, increased contents of chlorophyll a, total chlorophyll and carotenoids, enhanced accumulation of O_2_^−^, as well as heightened activities of superoxide dismutase, peroxidase, and catalase. Transcriptome profile analyses suggested that a total of 363 and 858 differentially expressed genes were obtained in the unifoliate leaves of adzuki bean seedlings treated with 32.5 mmol/L and 65.0 mmol/L NaCl groups, respectively. The up-regulated genes were mainly enriched in the spliceosome pathway, while the down-regulated genes were mainly enriched in pathways of plant hormone signal transduction, plant–pathogen interaction, and the MAPK signaling pathway in plants. These results provide new insight into exploring the response mechanisms of adzuki beans to salinity stress via transcriptome analyses.

## 1. Introduction

Salinity is a significant abiotic stress with a major impact on crop development and productivity, especially in arid and semi-arid regions [1]. It is estimated that approximately 20% of cultivated land and nearly 50% of irrigated land are affected by this stress in the whole world [2,3]. Consequently, salinity is considered a worldwide problem that is adverse to the sustainable development of modern agriculture [4]. The salinization of soil and water is mainly attributed to the accumulation of cations (Na^+^, Ca^2+^ and Mg^2+^) and anions (Cl^−^, SO_4_^2−^ and HCO_3_^−^) during natural processes and anthropogenic activity [5], and excessive salts in the soil solution induce osmotic stress, ion toxicity, and secondary stresses such as oxidative damage to hamper seed germination, plant growth, photosynthesis, and transpiration [5,6]. In severe cases, failure of seed set could be caused by reduced viability of pollen under salinity conditions [7]. Throughout evolution, plants have developed sophisticated strategies of adaptation to respond to salt stress. These strategies include (i) selective accumulation or exclusion of ions at the cellular and whole-plant levels, (ii) modulation of ion uptake and transport from roots to photosynthetic organs, (iii) synthesis of compatible solutes (osmolytes), (iv) changes in photosynthetic pathway and membrane structure, and (v) induction of hormonal and antioxidative responses [8].

Transcription is the first and most pivotal step in the modulation of gene expression [4], while transcriptome sequencing technology is an effective method to explore the transcriptional profiles of tissues, organs, and even single cells under specific conditions [9]. To date, many plant species have been studied in hundreds, if not thousands, of studies on salinity stress-related transcriptomes via the high-throughput sequencing technology, which facilitates the identification of target genes related to salinity stress responses. For example, a comprehensive analysis of differentially expressed genes (DEGs) in salt-treated soybean seedlings with a time-course experiment revealed that several hub genes involved in phytohormone signaling pathways and reprogramming of genes related to carbon and nitrogen played vital roles in soybean survival under salt stress [10]. Similarly, transcriptomic profiling of a salt-tolerant yardlong bean (*Vigna unguiculata* ssp. *sesquipedalis*) variety, Suzi 41, under high salinity, showed significant enrichment of cysteine/methionine metabolism pathway, in which 1-aminocyclopropane-1-carboxylate synthase, functioning in ethylene synthesis, was significantly up-regulated, suggesting a potential role of ethylene signaling pathway in mediating salt stress tolerance in *Vigna unguiculata* ssp. *sesquipedalis* [11]. These findings indicate that phytohormone signaling pathways are critically involved in salt stress tolerance, although plant species could utilize multipronged ways to overcome salinity stress.

Adzuki bean (*Vigna angularis* (Willd.) Ohwi & H. Ohashi) is a diploid legume with 22 chromosomes (2n = 2x = 22) from the *Fabaceae* (*Leguminosae*) family, and is also commonly known as azuki bean, aduki bean, small bean, red bean, and red mung bean. This legume is native to China and cultivated in more than 30 countries worldwide, predominantly in East Asia, including China, Korea, and Japan [12,13]. Adzuki beans can be used to make desserts, porridge, noodles, cakes, jelly, ice cream, and so forth, due to the abundance of high-quality protein, carbohydrates, polyphenols (especially flavonoids), and minerals [14,15]. Generally, the majority of landrace and improved adzuki bean varieties have a uniform red appearance, but other colors, including black, brown, white, beige, gold, green, and motley, also exist; these might be attributed to the differences in flavonoid biosynthesis [15,16]. As a traditional medicine, adzuki beans have been used to treat diseases such as edema, diarrhea, vomiting, and others in China, which was documented in the classical pharmacopoeia Ben Cao Gang Mu by Li Shizhen (an outstanding medical expert) in the Ming Dynasty [17,18]. China is the major output and export country of adzuki beans worldwide, with an annual yield of 250–350 thousand tons and export volume of 50–80 thousand tons, respectively [19]. However, its yield remains lower than that of staple food crops like wheat, rice, and maize. Furthermore, abiotic and biotic factors affect the growth of adzuki beans and can even result in yield loss [13]. For instance, the rainfall pattern change resulted in a 21.7% yield reduction in adzuki beans in Korea in 2018 [20].

Adzuki beans are classified as glycophytes sensitive to salt stress [13,21], while different varieties show varied levels of salt stress sensitivity. JP81144 and JP226893 were the most tolerant to salt stress in soil culture and hydroponic culture among the selected test varieties of adzuki beans, but both varieties exhibited lower salt tolerance compared with their cross-compatible wild relatives of Tojinbaka and Ukushima [22]. Salinity stress not only inhibited the germination of adzuki beans [13,23] but also affected the physicochemical parameters such as phenolic content and photosynthetic activity [21,24]. Meanwhile, several genes encoding basic leucine zipper (bZIP) transcription factors (TFs) and ethylene-response factor 3 (ERF3) were significantly modulated by salinity stress [25,26,27]. To date, studies on salinity stress on adzuki bean mainly focus on physicochemical changes and some simple regulators. Nevertheless, comprehensive transcriptomic analyses delineating salinity-responsive molecular mechanisms in adzuki bean seedlings have not been systematically characterized through an RNA-seq approach.

Therefore, in the current study, different concentrations of NaCl solution were designed to simulate salinity stress to treat adzuki bean seedlings based on our previous report [13]. Jin Xiao Dou 6 (JXD6), one cultivar from Shanxi province of China possessing potential stress tolerance and high yield, was subjected to salinity stress to improve the understanding of the genetic control related to salinity responses through jointly analyzing physicochemical indicators and transcriptomic data. The main metabolic pathways responsive to the designed stress were identified by transcriptome analysis. This study will lay the foundation for deepening the molecular mechanism of adzuki bean adaptation to salinity stress.

## 2. Results

### 2.1. Morphological Analysis of JXD6 Seedlings in Response to Salinity Stress

**Figure 1:**
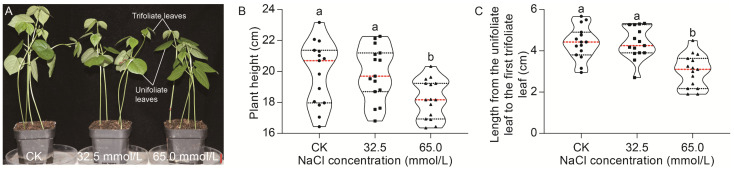
The effects of 0.0 mmol/L (CK), 32.5 mmol/L, and 65.0 mmol/L NaCl concentrations on the morphological changes in JXD6 seedlings. (**A**) Morphology of JXD6 seedlings. Bar (red), 2 cm; (**B**) Plant height; (**C**) Length from the unifoliate leaf to the first trifoliate leaf. Black dashed lines indicated quartiles; red dashed lines indicated the median. Different lowercase letters indicated statistical differences (*p* < 0.05) as evaluated by Duncan’s multiple range test method.

The plant height, as well as the length from the unifoliate leaf to the first trifoliate leaf, was investigated under salinity stress and normal conditions. As shown in Figure 1A, the plant height of JXD6 seedlings decreased progressively with increasing NaCl concentrations. No significant difference in plant height was observed between 32.5 mmol/L NaCl concentration and CK (0 mmol/L or normal condition). However, a significant reduction in plant height at 65.0 mmol/L NaCl concentration was found, compared with that at normal conditions and salinity treatment of 32.5 mmol/L NaCl (Figure 1B). The length from the unifoliate leaf to the first trifoliate leaf showed a similar trend to that of plant height (Figure 1C).

### 2.2. Physicochemical Analysis of JXD6 Seedlings in Response to Salinity Stress

#### 2.2.1. Effect of Salinity Stress on the Chloroplast Pigment Contents of Unifoliate Leaves from JXD6 Seedlings

**Figure 2:**
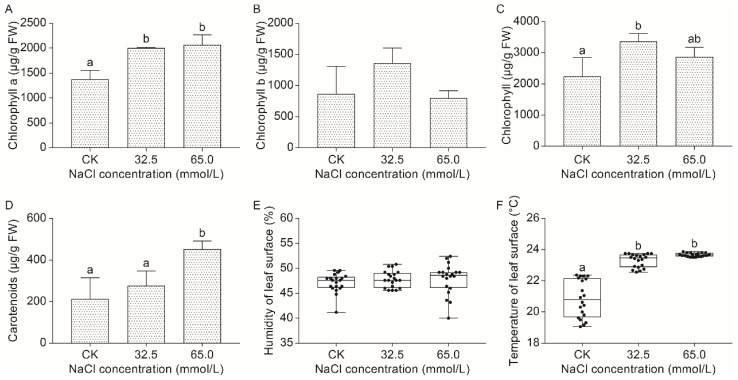
The effects of 0.0 mmol/L (CK), 32.5 mmol/L, and 65.0 mmol/L NaCl treatments on the chloroplast pigment contents of unifoliate leaves from JXD6 seedlings. (**A**) C_a_ content; (**B**) C_b_ content; (**C**) Total chlorophyll content. (**D**) C_tc_ content; (**E**) Leaf surface humidity; (**F**) Leaf surface temperature. Different lowercase letters indicated statistical differences (*p* < 0.05) as evaluated by Duncan’s multiple range test method.

Plant chlorophyll contents are considered an indicator of photosynthetic capacity. In the unifoliate leaves of JXD6 seedlings, chlorophyll a (C_a_) levels increased significantly under both 32.5 mmol/L and 65.0 mmol/L salinity treatments compared with those at normal conditions, although no significant difference was observed between the two salinity-treated groups (Figure 2A). At the same time, chlorophyll b (C_b_) showed no statistical difference among any of the groups (Figure 2B). Meanwhile, C_a_/C_b_ exhibited no statistical difference between CK and salinity treatment groups, although significant differences were observed under 32.5 mmol/L and 65.0 mmol/L NaCl concentrations (Figure S1). Total chlorophyll showed an initial increasing and then decreasing trend, although no significant difference was presented between 32.5 mmol/L and 65.0 mmol/L salinity treatment groups (Figure 2C). Interestingly, total carotenoids (C_tc_) exhibited an overall increasing trend under salinity stress, although no significant difference was detected between the normal condition and 32.5 mmol/L salinity stress (Figure 2D).

#### 2.2.2. Effect of Salinity Stress on the Surface Humidity and Temperature of Unifoliate Leaves from JXD6 Seedlings

For surface humidity of unifoliate leaves, seedlings from salinity-treated and normal groups showed no significant difference (Figure 2E). Intriguingly, salinity stress significantly increased the leaf surface temperature of unifoliate leaves compared with the CK group, although no difference was found between the 32.5 mmol/L and 65.0 mmol/L salinity treatment groups (Figure 2F).

#### 2.2.3. Effect of Salinity Stress on the Reactive Oxygen Species (ROS) and Membrane Permeability of Unifoliate Leaves from JXD6 Seedlings

**Figure 3:**
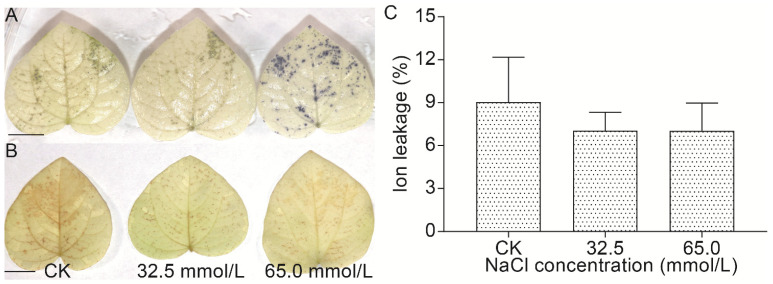
The effects of 0.0 mmol/L (CK), 32.5 mmol/L, and 65.0 mmol/L NaCl treatments on the ROS accumulation and ion leakage of unifoliate leaves from JXD6 seedlings. (**A**) O_2_^−^ staining of unifoliate leaves. Bar, 1 cm; (**B**) H_2_O_2_ staining of unifoliate leaves. Bar, 1 cm; (**C**) The examination of ion leakage.

ROS (H_2_O_2_ and O_2_^−^) accumulation in the unifoliate leaves from salinity-treated and untreated JXD6 seedlings was assessed histochemically by nitro-blue tetrazolium (NBT) and 3,3-diaminobenzidine (DAB) staining (Figure 3A,B). Compared with the CK group, the leaves from the 65.0 mmol/L salinity treatment group exhibited significantly higher O_2_^−^ accumulation (Figure 3A). However, H_2_O_2_ levels among the CK, 32.5 mmol/L, and 65.0 mmol/L salinity groups did not change significantly (Figure 3B). A similar pattern was observed in the ion leakage assay (Figure 3C), which suggested that membrane integrity was maintained under the designed NaCl concentrations.

#### 2.2.4. Effect of Salinity Stress on the Activities of Antioxidative Enzymes in Unifoliate Leaves from JXD6 Seedlings

**Figure 4:**
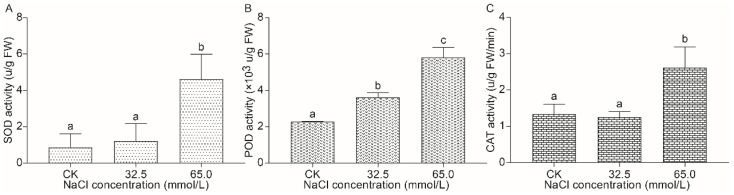
The activities of antioxidative enzymes in unifoliate leaves of JXD6 seedlings. (**A**) SOD activity; (**B**) POD activity; (**C**) CAT activity. Different lowercase letters indicated statistical differences (*p* < 0.05) as evaluated by Duncan’s multiple range test method.

In order to explore the antioxidant capacity of unifoliate leaves of JXD6 seedlings under different salinity stresses, the superoxide dismutase (SOD), peroxidase (POD), and catalase (CAT) activities were examined. The results showed that there were no differences in SOD and CAT activities in the 32.5 mmol/L salinity stress between the CK and treatment, while POD activity was significantly higher than that in unstressed seedlings (Figure 4). However, 65.0 mmol/L salinity treatment of JXD6 seedlings significantly increased SOD, POD, and CAT activities in the unifoliate leaves compared with those of 0 mmol/L NaCl-treated plants (Figure 4).

### 2.3. Quality Analysis of RNA Sequencing

To elucidate gene expression changes in unifoliate leaves of JXD6 seedlings under different NaCl treatments, RNA sequencing was conducted for comparative analysis. Three biological replicates were examined for each salinity stress (Table 1). A total of 396.13 M raw reads were generated from nine samples. After removing low-quality reads and adaptor sequences, approximately 382.28 M clean reads were obtained, and each sample was represented by over 6.32 Gb clean bases. The range of Q20 and Q30 values was 97.62–97.98% and 93.41–94.37%. Out of total clean reads from nine samples, 82.51–88.10% and 74.07–78.30% were total and unique mapping, respectively. These results indicated that the RNA sequencing data were reliable in the present study.

### 2.4. Salinity Treatment Affects Gene Expression in Unifoliate Leaves of JXD6 Seedlings

**Figure 5:**
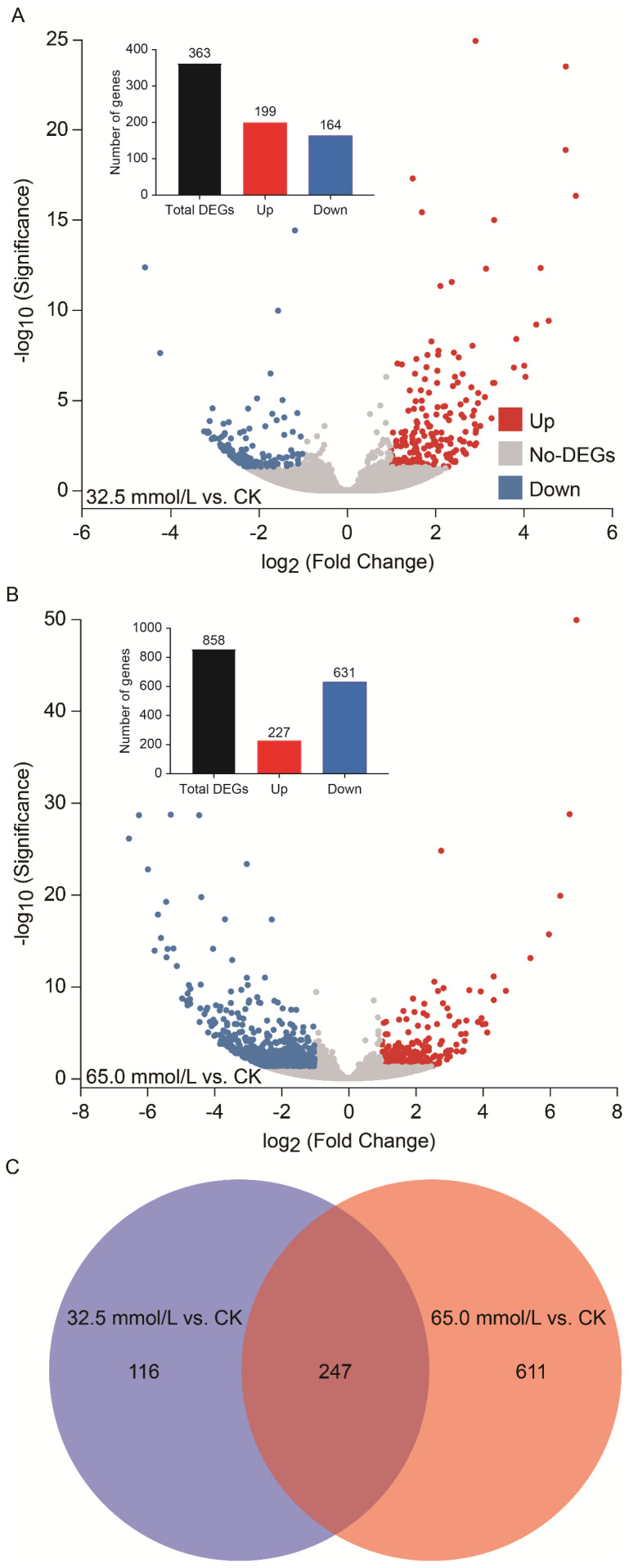
Overview of differentially expressed genes (DEGs) in the 32.5 mmol/L vs. CK and 65.0 mmol/L vs. CK comparisons. (**A**) Number of DEGs in the 32.5 mmol/L vs. CK comparison; (**B**) Number of DEGs in the 65.0 mmol/L vs. CK comparison; (**C**) Venn diagram analysis of overlapping and non-overlapping DEGs among the two salinity-treated groups compared with CK. The DEGs were obtained by *p* < 0.05 and |log_2_Fold Change| ≥ 1.

In comparison with the CK group, significant changes in gene expression (*p* < 0.05 and |log_2_Fold Change| ≥ 1) were detected in response to salinity stress. Specifically, 363 DEGs were identified under 32.5 mmol/L salinity stress, comprising 199 up-regulated and 164 down-regulated genes (Figure 5A). Under 65.0 mmol/L salinity stress, a total of 858 DEGs were examined, of which 227 were up-regulated, and 631 were down-regulated (Figure 5B). Among the DEGs in the two salinity-treated groups, 247 genes were expressed significantly in common (Figure 5C), while 116 and 611 genes were only expressed significantly in 32.5 mmol/L and 65.0 mmol/L salinity conditions, respectively. Furthermore, the number of DEGs increased markedly with rising NaCl concentrations designed in this study (Figure 5).

### 2.5. Gene Ontology (GO) Analyses of Differential Expression of Genes

**Figure 6:**
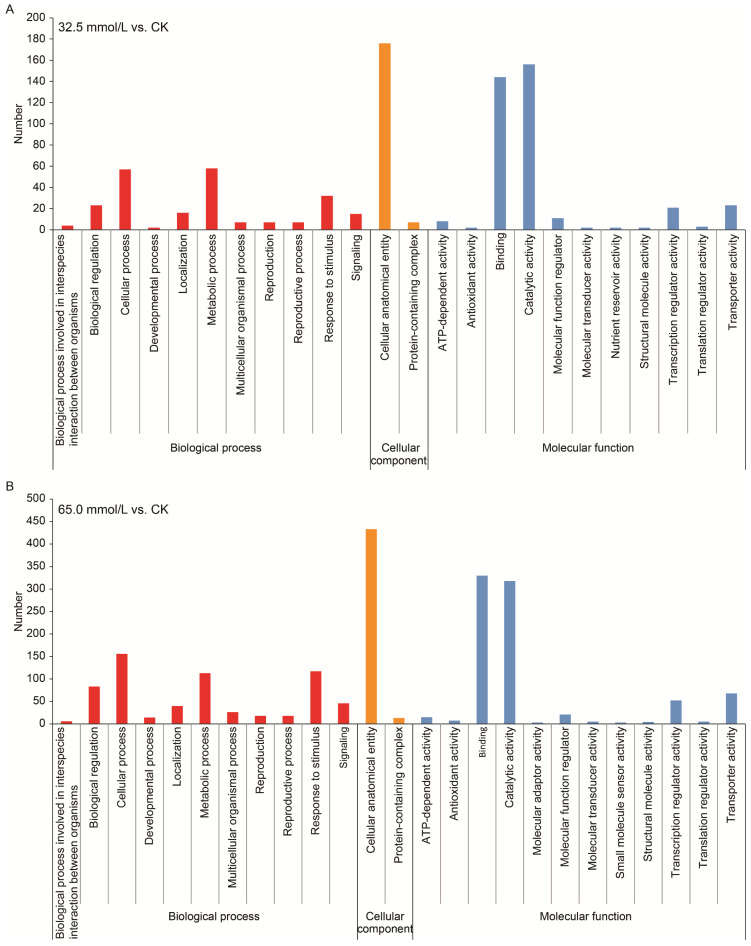
Gene ontology (GO) classification of the differentially expressed genes (DEGs) in the two compared groups. (**A**) 32.5 mmol/L vs. CK comparison; (**B**) 65.0 mmol/L vs. CK comparison.

GO analysis was conducted for the functional classification of DEGs. A total of 24 and 25 GO classes/terms were, respectively, obtained from the two salinity-treated groups and assigned to three categories, viz., biological process, cellular component, and molecular function (Figure 6). In the category of biological process, “cellular process”, “response to stimulus”, and “metabolic process” were prominently represented in both treatment groups, while “catalytic activity”, “binding”, and “transporter activity” were the most highly represented classes/terms in the molecular function classification.

**Figure 7:**
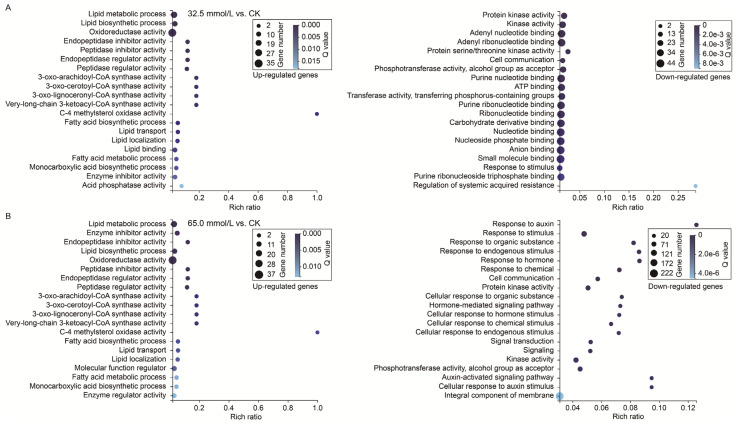
Gene ontology (GO) enrichment analysis of the differentially expressed genes (DEGs) in the two compared groups. (**A**) 32.5 mmol/L vs. CK comparison; (**B**) 65.0 mmol/L vs. CK comparison.

To further understand the main functional characteristics of these DEGs, GO enrichment analysis (*p* < 0.05) was performed with the annotated genes in the unifoliate leaves of JXD6 seedlings under two salinity treatments. The top 20 terms enriched for the up- and down-regulated DEGs in the designed NaCl conditions are shown in Figure 7. In the 32.5 mmol/L vs. CK comparison, the GO terms related to “lipid metabolic process”, “lipid biosynthetic process”, and “oxidoreductase activity” were significantly enriched in the up-regulated DEGs, and the three significantly enriched terms for the down-regulated DEGs were “protein kinase activity”, “kinase activity”, and “adenyl nucleotide binding” (Figure 7A). In the 65.0 mmol/L vs. CK comparison, the significantly enriched GO terms were “lipid metabolic process”, “enzyme inhibitor activity”, and “endopeptidase inhibitor activity” in the up-regulated DEGs, and “response to auxin”, “response to stimulus”, and “response to organic substance” in the down-regulated DEGs (Figure 7B). While based on the number of enriched DEGs in the top 20 GO terms, the three terms with maximum numbers of up-regulated DEGs in both the two comparisons were “oxidoreductase activity”, “lipid metabolic process”, and “lipid biosynthetic process”, and those of down-regulated DEGs were “small molecule binding”, “nucleotide binding”, and “nucleoside phosphate binding” in the 32.5 mmol/L vs. CK comparison and “integral component of membrane”, “response to stimulus”, and “kinase activity” in the 65.0 mmol/L vs. CK comparison.

### 2.6. Kyoto Encyclopedia of Gene and Genome (KEGG) Analyses of Differentially Expressed Genes

**Figure 8:**
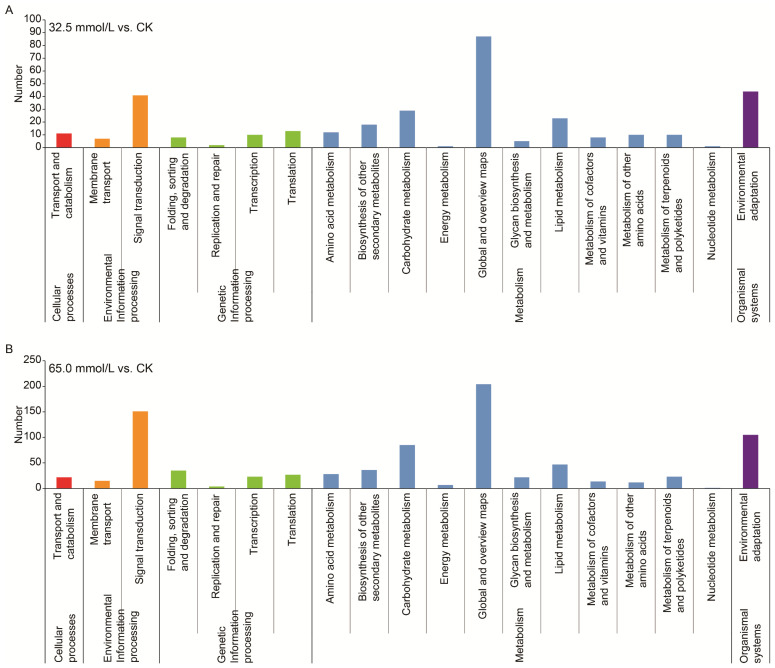
Kyoto Encyclopedia of Genes and Genomes (KEGG) pathway classifications of the differentially expressed genes (DEGs) in the two compared groups. (**A**) 32.5 mmol/L vs. CK comparison; (**B**) 65.0 mmol/L vs. CK comparison.

A comprehensive exploration of the DEGs using the KEGG databases was performed to understand the changes in biological pathways. According to the KEGG annotation, both DEGs from the 32.5 mmol/L vs. CK comparison and 65.0 mmol/L vs. CK comparison were assigned to five categories, namely, cellular process, environmental information processing, genetic information processing, metabolism, and organismal systems (Figure 8). Most of the annotated genes were involved in the “global and overview maps”, “signal transduction”, “environmental adaptation”, “carbohydrate metabolism”, and “lipid metabolism” (Figure 8).

**Figure 9:**
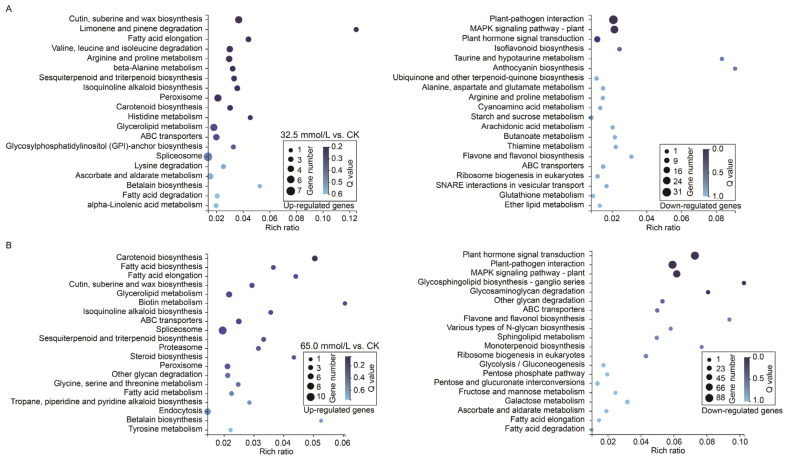
Kyoto Encyclopedia of Genes and Genomes (KEGG) pathway enrichment analysis of the differentially expressed genes (DEGs) in the two compared groups. (**A**) 32.5 mmol/L vs. CK comparison; (**B**) 65.0 mmol/L vs. CK comparison.

To further elucidate the functional implications of the DEGs, KEGG pathway enrichment analysis was subsequently performed. The results revealed that the up-regulated genes in 32.5 mmol/L vs. CK comparison were commonly enriched in pathways such as “spliceosome”, “cutin, suberine, and wax biosynthesis”, and “peroxisome” (Figure 9A), while that in 65.0 mmol/L vs. CK comparison were enriched in pathways, including “spliceosome”, “endocytosis”, and “glycerolipid metabolism” (Figure 9B). Notably, the most enriched pathway of the up-regulated genes under salinity stress was “spliceosome”. Interestingly, the down-regulated genes in the 32.5 mmol/L vs. CK comparison and 65.0 mmol/L vs. CK comparison were both enriched in the pathways represented by “plant hormone signal transduction”, “plant–pathogen interaction”, and “MAPK signaling pathway-plant” (Figure 9).

### 2.7. Differentially Expressed Genes Related to Spliceosome Pathway

**Figure 10:**
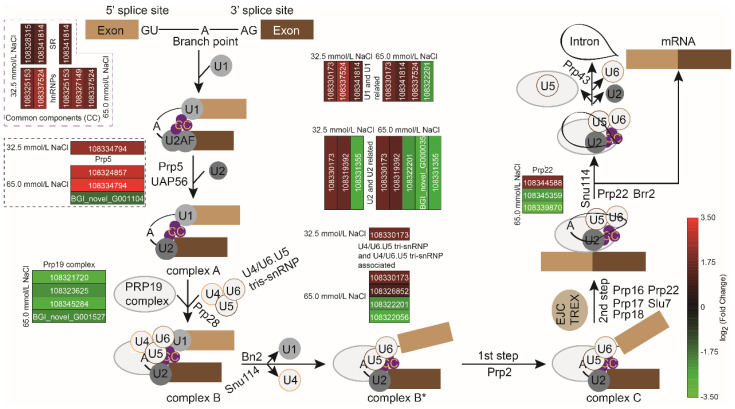
Mapping of altered transcripts related to spliceosome pathway during 32.5 mmol/L and 65.0 mmol/L salinity treatments.

According to Figure 10 and Table S1, ten genes associated with spliceosome components and splicing factors, including U1, U2, U4/U6.U5 tri-snRNP, and their related genes, common components of hnRNPs, SR, and Prp5, increased significantly under 32.5 mmol/L salinity treatment, while one gene encoding SF3b in U2 and U2-related genes showed significant down-regulation. With NaCl concentration increasing to 65.0 mmol/L, a significant number of up- (14) and down-regulated (13) genes were observed in the spliceosome pathway, compared with the 32.5 mmol/L salinity group. Among them, the splicing factor of Prp19 complex-related genes showed significant down-regulation (Figure 10 and Table S1). These findings suggested that salinity treatments enhanced the post-transcriptional regulation mediated by the spliceosome in the JXD6 seedlings.

### 2.8. Differentially Expressed Genes Related to Signal Transduction of Plant Hormones

**Figure 11:**
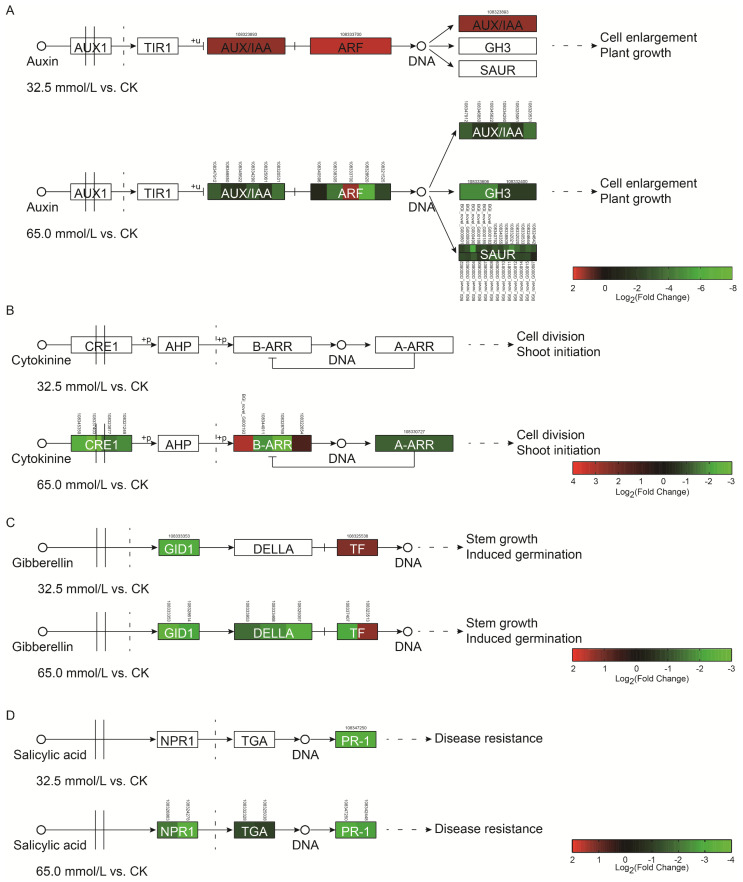
Differentially expressed genes (DEGs) involved in auxin (**A**), cytokinin (**B**), gibberellin (**C**), and salicylic acid (**D**) signal transduction in salinity-treated adzuki bean seedlings. AUX/IAA: *auxin/indoleacetic acid*; ARF: *auxin response factor*; GH3: *Gretchen Hagen 3*; SAURs: *small auxin-up RNA genes*; CRE1: *cytokinin receptor*; A-ARRs: *type-A arabidopsis response regulators*; B-ARRs: *type-B arabidopsis response regulators*; GID1: *GA-insensitive dwarf1*; DELLA: *GA response repressor*; TF: *transcription factor*; NPR1: *non-expressor of PR1*; TGA: *TGA motif-binding protein*; PR-1: *pathogenesis-related protein 1*.

Among DEGs, 2 and 40 genes involved in auxin signaling transduction were examined in the 32.5 mmol/L vs. CK comparison and the 65.0 mmol/L vs. CK comparison, respectively. Only two genes related to *auxin/indoleacetic acid* (*AUX/IAA*) and *auxin response factor* (*ARF*) were significantly up-regulated during 32.5 mmol/L NaCl treatment (Figure 11A and Table S2). When the NaCl concentration increased to 65.0 mmol/L, 39 genes involved in *AUX/IAA*, *ARF*, *small auxin-up RNA genes* (*SAURs*), and *Gretchen Hagen 3* (*GH3*) were significantly down-regulated, except one gene associated with *ARF* (Figure 11A and Table S2). These results indicated that the growth of adzuki bean seedlings might be inhibited with an increase in NaCl concentrations.

In the cytokinin (CK) responsive pathway, no gene was identified to express differently under 32.5 mmol/L NaCl treatment. However, nine genes showed significantly different expression in the 65.0 mmol/L vs. CK comparison. Among these, seven genes encoding cytokinin receptor (CRE1), type-A *Arabidopsis* response regulators (A-ARRs), and B-ARRs were significantly down-regulated, and two genes involved in B-ARRs were significantly up-regulated (Figure 11B and Table S3). These results suggested that cell division in the adzuki bean seedlings might be weakened with the increasing NaCl concentrations.

In the gibberellin (GA) signaling pathway, two and seven genes in the 32.5 mmol/L vs. CK comparison and 65.0 mmol/L vs. CK comparison were identified during the salinity stress, respectively. Two genes related to *GA-insensitive dwarf1* (*GID1*) and *transcription factor* (*TF*) were down- and up-regulated in the 32.5 mmol/L vs. CK comparison, respectively (Figure 11C and Table S4). Intriguingly, six genes encoding GID1, GA response repressor (DELLA), and TF showed significantly down-regulated in the 65.0 mmol/L vs. CK comparison, and only one TF-encoding gene was significantly up-regulated (Figure 11C and Table S4). These results suggested that the stem growth of adzuki bean was also affected by the salinity treatment.

In the salicylic acid (SA) signaling pathway, only one gene encoding non-expressor of PR1 (NPR1) showed significant down-regulation in the 32.5 mmol/L vs. CK comparison, while six genes involved in *NPR1*, *TGA motif-binding protein* (*TGA*), and *pathogenesis-related protein 1* (*PR-1*) were significantly down-regulated by 65.0 mmol/L NaCl treatment (Figure 11D and Table S5). These results suggested that salinity inhibited the gene expression of SA signaling transduction in the adzuki bean seedlings.

Additionally, abscisic acid (ABA), ethylene, and brassinosteroid (BR) signaling pathways were also analyzed during the salinity treatment. Three genes, including two down-regulated genes encoding ABA receptor PYR/PYL and one up-regulated gene encoding protein phosphatase 2C (PP2C), showed varied expression levels in the ABA signaling pathway under both salinity stresses (Figure S2A and Table S6). In the ethylene signaling pathway, one gene encoding ethylene-responsive transcription factor 1/2 (ERF1/2) showed down-regulation in both the 32.5 mmol/L vs. CK comparison and the 65.0 mmol/L vs. CK comparison, while seven genes involved in *mitogen-activated protein kinase kinase 4/5* (*SIMKK*) were down-regulated specifically in the 65.0 mmol/L vs. CK comparison (Figure S2B and Table S6). In BR signaling pathway, 8 genes related to *brassinosteroid insensitive 1-associated receptor kinase 1* (*BAK1*, 5 genes) and *protein brassinosteroid insensitive 1* (*BRI1*, 3 genes) showed significant down-regulation in the 32.5 mmol/L vs. CK comparison, while 18 genes encoding BAK1 (9 genes)/BRI1 (6 genes), BR-signaling kinase (BSK, 2 genes) and xyloglucan:xyloglucosyl transferase TCH4 (TCH4, 1 gene) were significantly down-regulated with the exception of one gene associated with *BRI1* (Figure S2C and Table S6). Overall, genes of plant hormone signaling pathways were significantly down-regulated in the adzuki bean seedlings with the increase in salt concentrations.

### 2.9. Differentially Expressed Genes Related to Plant–Pathogen Interaction and Mitogen-Activated Protein Kinase (MAPK) Signaling Pathways

A total of forty DEGs annotated to plant–pathogen interaction pathways were identified in the 32.5 mmol/L vs. CK comparison (Table S7). Among these, 31 genes showed significant down-regulation, and only 9 genes were significantly up-regulated (Table S7). When salt concentration increased to 65.0 mmol/L, the number of DEGs involved in plant–pathogen interaction pathways increased to 100 genes, of which 89 were significantly down-regulated, and only 11 were significantly up-regulated (Table S7). These results indicated that salinity stress might limit the expression of plant–pathogen interaction pathway-related genes with the increase in salt concentrations.

A substantial number of DEGs involved in MAPK signaling pathways were also identified in both the 32.5 mmol/L vs. CK comparison and the 65.0 mmol/L vs. CK comparison (Table S8). In the 32.5 mmol/L vs. CK comparison, 34 genes were detected in the leaves of adzuki bean seedlings, of which 25 were significantly down-regulated, and 9 were significantly up-regulated. While in the 65.0 mmol/L vs. CK comparison, 80 genes were found to have varied expressions; notably, 72 were significantly down-regulated, and only 8 were significantly up-regulated. These results also suggested that the increase in NaCl concentrations might limit the gene expression of MAPK signaling pathways.

### 2.10. Validation of Gene Expression Patterns in Transcriptomic Data

**Figure 12:**
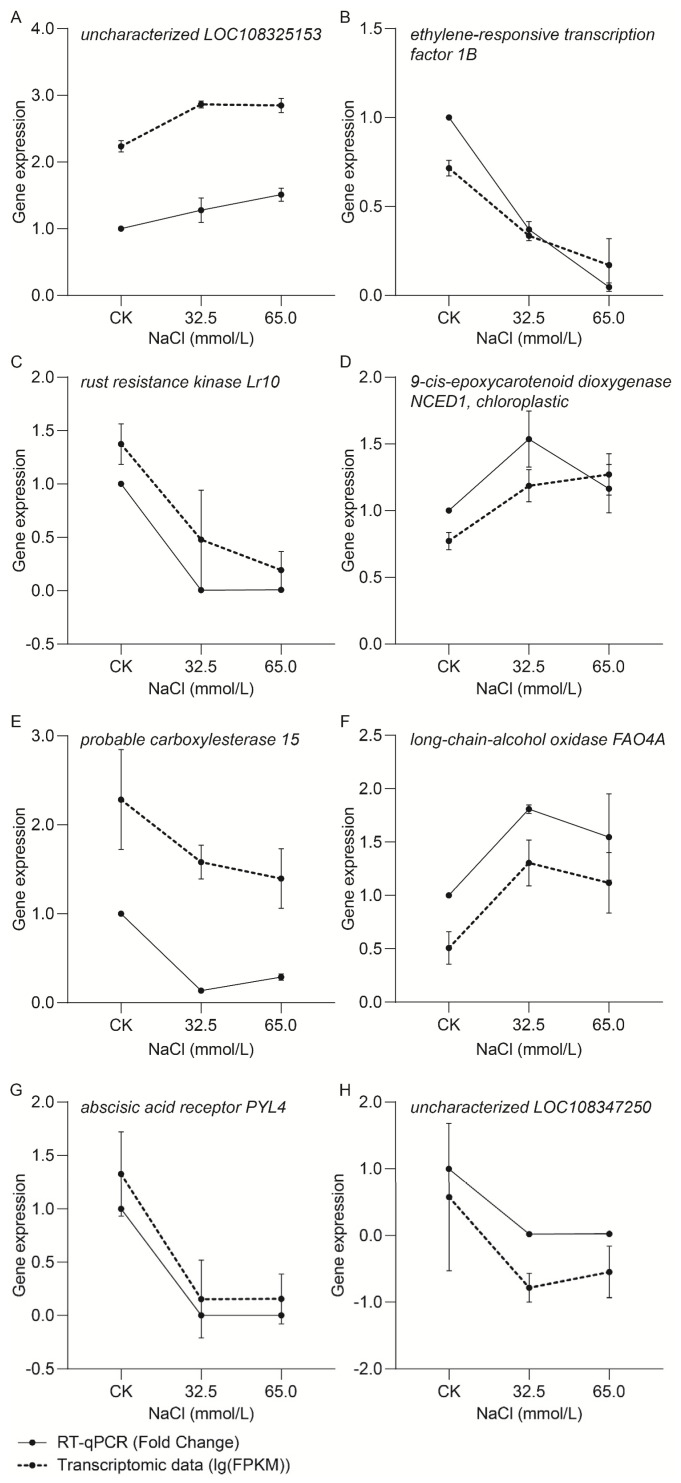
RT-qPCR validation of transcriptomic data with eight randomly selected genes. (**A**) Gene expression of *uncharacterized LOC108325153*; (**B**) Gene expression of *ethylene-responsive transcription factor 1B*; (**C**) Gene expression of *rust resistance kinase Lr10*; (**D**) Gene expression of *9-cis-epoxycarotenoid dioxygenase NCED1, chloroplastic*; (**E**) Gene expression of *probable carboxylesterase 15*; (**F**) Gene expression of *long-chain-alcohol oxidase FAO4A*; (**G**) Gene expression of *abscisic acid receptor PYL4*; (**H**) Gene expression of *uncharacterized LOC108347250*. Values represent the mean ± SD of three biological replicates.

To verify the reliability of transcriptomic data, eight genes in the leaves of the adzuki bean plant were randomly selected for RT-qPCR validation. The relative expression levels (lg(FPKM)) in the transcriptomic data and RT-qPCR results (Fold Change) of the selected genes are shown in Figure 12. The expression patterns of these genes showed a fairly good match between transcriptomic data and RT-qPCR results, indicating that the quality of transcriptomic data is trustworthy.

## 3. Discussion

Generally, salinity stress can inhibit the growth of plants and reduce plant height [28,29], but several studies have shown that low concentrations of salt can indeed stimulate the growth of some plants [30,31]. In this study, when the 14-day adzuki bean seedlings were treated with 32.5 mmol/L NaCl treatment for 7 days, no significant differences were found in the length from the unifoliate leaf to the first trifoliate leaf and plant height, while a significant reduction in these traits was observed in the 65.0 mmol/L NaCl group (Figure 1). Therefore, the inhibition of plant height during salinity stress required a threshold of salt concentration. Plant height is closely associated with cell division, elongation, and differentiation, which are modulated by different internal and external factors. Signal transduction of plant hormones plays a critical role in cell fate progression [32] and directly or indirectly regulates plant height. Existing research has demonstrated that plant heights of *Sesamum indicum*, *Capsicum baccatum,* and *Pinus yunnanensis* were regulated by the plant hormone signal transduction pathways [33,34,35]. During salinity stress, an abundance of genes related to plant hormone signal transduction pathways were differentially expressed in leaves of adzuki bean seedlings, especially those involved in auxin, CK, and GA signal transduction pathways, which are major regulators of cell division, enlargement, and stem elongation [36,37,38]. In the 32.5 mmol/L vs. CK comparison, only four genes, two up-regulated genes of the auxin signal transduction pathway and two genes (one up-regulated and one down-regulated) of the GA signal transduction pathway, were significantly modulated by the salinity stress (Figure 11A,C and Tables S2 and S4). The up-regulation of the three genes in the adzuki bean seedlings might play vital roles in the maintenance of plant height under 32.5 mmol/L NaCl treatment, which resulted in unaltered morphology of length from the unifoliate leaf to the first trifoliate leaf and plant height (Figure 1). However, DEGs were significantly increased to 56 in 65.0 mmol/L vs. CK comparison (Figure 11A–C and Tables S2–S4). Intriguingly, 40 DEGs were involved in the auxin signal transduction pathway among the 56 genes, and surprisingly, 27 DEGs encoding SAUR proteins were down-regulated (Figure 11A and Table S2). This gene family plays an important role in the regulation of plant dynamic and adaptive growth, and overexpression of *SAURs* can induce cell elongation in Arabidopsis at the cell level, and growth of leaves and stems at the whole plant level in the majority of studies [39,40]. In the *Atropa belladonna*, overexpression of *AbSAUR1* showed increased plant height [41], whereas knockout of *OsSAUR10* resulted in a dwarf phenotype in rice plants [42]. The reduced plant height observed in adzuki bean seedlings under 65.0 mmol/L NaCl might, therefore, be directly or indirectly attributable to the down-regulation of the auxin signal transduction pathway. Meanwhile, the CK and GA signal transduction pathways might also contribute to the decreased length from the unifoliate leaf to the first trifoliate leaf and plant height due to the down-regulated genes and cross-talk with the auxin signal transduction pathway (Figure 1). Additionally, one gene encoding TCH4 was down-regulated in the BR signal transduction pathway, and might influence cell elongation (Figure S2). However, knockout of *TCH4* led to no alteration of plant height in Arabidopsis [43].

Chlorophyll plays a vital role in the absorption of light energy during photosynthesis. However, chlorophyll content was decreased in many plants exposed to salinity stress [44,45]. In this study, both salinity treatment groups showed an increase in C_a_ content compared with the CK group, while no difference was detected in C_b_ content among the three groups (Figure 2A,B). Considering the total chlorophyll content, the 32.5 mmol/L NaCl group significantly increased the chlorophyll content among the three groups, and the 65.0 mmol/L NaCl group showed no difference from the CK and the 32.5 mmol/L NaCl groups (Figure 2C). The similar results were reported by the studies on sorghum cultivar Extra Sweet and Turkish tobacco variety Akhisar 97 treated with salinity stresses, which suggested that low salinity stress (50 mmol/L NaCl) increased C_a_ and total chlorophyll content, while no significant change was found in C_b_ content [46,47]. Additionally, other investigations also reported the increase in total chlorophyll content under low salinity stress [48]. Carotenoids are the second most abundant pigments that occur naturally and are crucial for harvesting light during photosynthesis and acting as antioxidants in mitigating the generation of reactive molecules, especially ROS [49]. With rising NaCl concentrations, total carotenoid content increased significantly in the unifoliate leaves of JXD6 seedlings (Figure 2D). Meanwhile, the activities of SOD, POD, and CAT were also enhanced, especially in the 65.0 mmol/L NaCl group (Figure 4). At the transcriptional level, the GO term of “oxidoreductase activity” and pathway of carotenoid biosynthesis were significantly enriched during the GO and KEGG enrichment analysis of up-regulated DEGs in the salt-treated group, respectively (Figure 7 and Figure 9). These results suggested that adzuki bean seedlings enhanced the antioxidant capability to scavenge ROS to protect plant growth and development without oxidative damage during the salinity stress. The histochemical examination of O_2_^−^ and H_2_O_2_ accumulation suggested that the 65.0 mmol/L NaCl group showed high O_2_^−^ content, and H_2_O_2_ content exhibited no differences among the three groups (Figure 3A,B). The increases in total carotenoid content and SOD activity correlated positively with the accumulation of O_2_^−^, and the elevated POD and CAT activities, along with total carotenoids, contributed to H_2_O_2_ homeostasis in the unifoliate leaves of JXD6 seedlings during the salinity stress at the designed NaCl concentration in this study. The ion leakage assay further supported the maintenance of ROS homeostasis in the unifoliate leaves of adzuki bean seedlings (Figure 3C). Several studies suggested that salinity stress could significantly enhance the activities of antioxidant enzymes [50,51,52] and increase the content of total carotenoids [52,53] within a defined range of NaCl concentrations. Meanwhile, the GO term of “oxidoreductase activity” was also enriched in the up-regulated DEGs of *Sonneratia caseolaris* and *Phragmites australis* treated with salinity stress [54,55], and the pathway of carotenoid biosynthesis was reported to be enriched in the up-regulated DEGs of salt-sensitive *indica* rice HuangHuaZhan during salinity stress [56].

Leaf surface temperature and humidity could serve as sensitive biophysical indicators of ambient environmental dynamics. The two indicators exhibited a consistent trend with circadian rhythm under the normal conditions [57,58,59]. However, when plants are subjected to stress, leaf surface temperature and humidity are rigorously modulated. Salinity stress, as well as water stress, could induce an increase in leaf surface temperature [59,60] and a decrease in leaf surface humidity [59]. Intriguingly, mechanical damage such as leaf detachment led to an increase in leaf surface temperature and humidity significantly due to the evaporation and drying of the leaf [59]. In this study, the leaf surface temperature of unifoliate leaves in JXD6 seedlings was significantly increased after salinity stress, although no difference was observed between 32.5 mmol/L and 65.0 mmol/L NaCl groups (Figure 2F), while leaf surface humidity showed little alteration in the adzuki bean seedlings among the three groups (Figure 2E). The results demonstrated that the leaf surface temperature of JXD6 seedlings exhibited heightened sensitivity to salinity stress, whereas the leaf surface humidity displayed comparatively lower responsiveness under such conditions in the current study. Previous studies suggested that temperature increase could induce frequency elevation of major modes of alternative splices (AS), especially retained introns and skipped exons [61]. The increase in leaf surface temperature was attributed to the salinity stress and might further induce the expression of genes related to the spliceosome pathway to modulate AS. With salt concentration increase, DEGs of spliceosome pathway were significantly increased (Figure 10 and Table S1), indicating that modulation of this pathway was enhanced through positive or negative feedbacks, which might result in increasing number of DEGs involved in AS (83 in the 32.5 mmol/L NaCl group, and 94 in the 65.0 mmol/L NaCl group) via elevation of leaf surface temperature to protect adzuki bean seedlings to adapt to salinity stress (Table S9).

Additionally, the MAPK signaling pathway and plant–pathogen interaction signaling pathway were significantly enriched in the unifoliate leaves of JXD6 seedlings during salinity stress (Figure 9). These pathways exhibit an interconnected regulatory architecture that enables them to adapt to environmental stimuli and pathogenic assaults [62]. Under salinity stress, cucumber seedlings exhibited a significantly higher number of down-regulated DEGs associated with the MAPK signaling pathway and plant–pathogen interaction signaling pathways compared to up-regulated DEGs in these ways [63]. Similar results were observed in the unifoliate leaves of JXD6 seedlings treated with salinity stress (Figure 9). Another report indicated contrary results that DEGs enriched in the two pathways were all up-regulated in the roots of salt-treated cotton (*Gossypium hirsutum*) at the seedling stage [64]. These findings demonstrate that divergent species employ distinct molecular regulatory mechanisms of signaling transduction pathways to adapt to salinity stress.

## 4. Materials and Methods

### 4.1. Plant Materials and Salt Treatment

Fully developed aduki bean seeds of cultivar JXD6 obtained from our laboratory were selected and planted in seedling pots (5 seeds/pot, volume of 300 mL) containing a mixture of nutrient soil (PINDSTRUP SUBSTRATE, particle sizes 0–10 mm, pH 5.5) and vermiculite (1:1 *v*/*v*). The seedling pots were placed in the growth chamber under a 16/8 h light (white light-emitting diode lamps (T8, PHILIPS, Cambridge, MA, USA), 100 μmol·m^−2^·s^−1^)/dark period at 23 °C ± 1 °C and watered with 100 mL distilled water every day. Thinning was performed to retain four seedlings per pot after 14 days, and 100 mL per pot of 0 mmol/L (normal condition, CK), 32.5 mmol/L, and 65.0 mmol/L NaCl solutions were watered to the different groups of seedlings continuously for 7 days. After salinity stress, unifoliate leaves from at least 12 21-day-old plants of each group were sampled, frozen with liquid nitrogen, and stored at −80 °C for subsequent determination of enzyme activity and RNA-seq analysis.

### 4.2. Seedling Growth Measurement

After 7 days of salinity stress, the plant height and length from the unifoliate leaf to the first trifoliate leaf were determined using a millimeter ruler. Fifteen plants from three biological replicates for each group (CK, 32.5 mmol/L and 65.0 mmol/L NaCl) were randomly selected for measurement to ensure the reliability and statistical significance of the results.

### 4.3. Photosynthetic Pigments Determination

The photosynthetic pigment content of adzuki bean seedlings was determined as previously described [65]. Briefly, 0.2 g of unifoliate leaves from each group of JXD6 seedlings was weighed and transferred into a 15 mL centrifuge tube filled with 10 mL 95% ethanol. Photosynthetic pigments were extracted under dark conditions for 48 h at room temperature. The absorbance was obtained by the UV-vis spectrophotometer (SPECORD^®^ 200 PLUS, Analytikjena, Jena, Germany) at the wavelengths of 664 nm, 649 nm, and 470 nm. The photosynthetic pigments concentration (μg/mL) was calculated using the following formulas: C_a_ = 13.36 × A_664_ − 5.19 × A_649_, C_b_ = 27.43 × A_649_ − 8.12 × A_664_, C_tc_ = (1000 × A_470_ − 2.13 × C_a_ − 97.63 × C_b_)/209. Photosynthetic pigment content (μg/g Fresh Weight, FW) = concentration × 10 mL × dilution ratio/sample weight (g). Three biological replicates were performed for each group.

### 4.4. Surface Humidity and Temperature of Unifoliate Leaves Measurement

The surface humidity and temperature of unifoliate leaves from JXD6 seedlings after salinity stress were determined using a plant nutrient analyzer (TYS-4N [66,67], Topo Yunnong Technology Co., Ltd., Hangzhou, China). Twenty plants from three biological replicates for each group were randomly selected for measurement.

### 4.5. Histochemical ROS Detection

The histochemical level of different ROS was detected according to the previous method [68]. For the detection of O_2_^−^, unifoliate leaves from JXD6 seedlings after salinity stress were immersed in a 1 mg/mL NBT solution (Solarbio, Beijing, China) until the dark blue color appeared and then transferred into 95% ethanol for decolorization until the leaves became white. For the detection of H_2_O_2_, the unifoliate leaves were immersed in 0.5 mg/mL DAB solution (Solarbio, Beijing, China) overnight, and then, 95% ethanol was used to decolor. Photographs were taken with a camera (Canon EOS 80D, Tokyo, Japan).

### 4.6. Ion Leakage Determination

Ion leakage of unifoliate leaves from JXD6 seedlings after salinity stress was measured using a conductivity meter (Mettler FE38, Zurich, Switzerland) with the method described by previous reports [69,70]. Unifoliate leaves (0.2 g) were thoroughly washed with running tap water and then with double-distilled water. Thereafter, the leaves were placed in 10 mL of double-distilled water at 40 °C for 30 min. The electrical conductivity was detected and recorded as C_1_ after the sample temperature dropped to room temperature. Subsequently, the abovementioned samples were placed in a boiling water bath for 10 min, cooled again to room temperature, and the electrical conductivity was measured as above and recorded as C_2_. The ion leakage was calculated by the following formula: ion leakage = [1 − (C_1_/C_2_)] × 100%. Three biological replicates were performed for each group.

### 4.7. Antioxidant Enzyme Activity Measurement

The activities of SOD, POD, and CAT were determined based on previous methods [71,72] with a SPECCORD^®^ 200 PLUS UV/visible Spectrophotometer (Jena, Germany). Unifoliate leaves (0.1 g) were ground into a homogenate in 4 mL of 50 mmol/L sodium phosphate buffer (pH 7.8). The homogenate was then centrifuged at 12,000· *g* for 10 min at 4 °C. SOD activity was determined by the NBT assay at a wavelength of 560 nm. POD activity was determined using the guaiacol colorimetric method at a wavelength of 470 nm. CAT activity was measured according to the degradation of H_2_O_2_ at a wavelength of 240 nm. Three biological replicates were performed for each group.

### 4.8. RNA-Seq Analysis

The procedures of RNA-seq experiments and data analysis, including RNA extraction, fragmentation, cDNA synthesis, adapter addition, PCR amplification, sequencing, and primary data processing for each sample, were performed by BGI (Shenzhen, China) with a DNBSEQ platform, which provides a high-throughput, high-quality sequencing necessary to capture and analyze gene expression data. Gene expression levels were compared between CK and 32.5 mmol/L NaCl groups, as well as between CK and 65.0 mmol/L NaCl groups, based on the FPKM (fragments per kilobase of transcript per million base pairs sequenced). The genes with a Q < 0.05, *p* < 0.05, and |log_2_Fold Change| ≥ 1 were considered significantly differentially expressed.

### 4.9. Functional Analysis of DEGs

The DEGs from two comparisons were analyzed with VennPainter software 1.2.0 [73]. GO and KEGG enrichment analyses were performed for DEGs using TermFinder and phyper in R 4.2.2 software, respectively. The corrected *p* < 0.05 was considered to be significantly enriched.

### 4.10. Real-Time Fluorescence Quantitative PCR (RT-qPCR) Verification of DEGs

For RT-qPCR, total RNA was extracted from unifoliate leaves of JXD6 seedlings treated with different concentrations of NaCl using the TransZol Up Plus RNA Kit (TransGen Biotech, Beijing, China); the reverse transcriptase system (TransScript^®^ One-Step gDNA Removal and cDNA Synthesis SuperMix, TransGen Biotech, Beijing, China) was used to synthesize the cDNA, and RT-qPCR was performed on a QuantStudioTM 3 Real-Time PCR Instrument (Thermo Fisher Scientific, Singapore) using PerfectStart^TM^ Green qPCR SuperMix (+Dye I/+Dye II, TransGen Biotech, Beijing, China) based on the instructions of manufacturer. For the verification of RNA-seq data, the relative expression levels of eight randomly selected genes were examined with three biological and three technical replicates for each group using the 2^−ΔΔCT^ method [74]. Actin was selected and used as a reference gene. The primers used for RT-qPCR are listed in Supplementary Materials Table S10.

### 4.11. Data Statistics and Analysis

The statistical analysis of significant differences among designed groups was conducted by IBM SPSS Statistics version 23.0 software (SPSS Inc., Chicago, IL, USA) with one-way analysis of variance (ANOVA) and Duncan’s multiple range test method. Different lowercase letters in figures indicated statistical differences (*p* < 0.05). GraphPad Prism version 8.0 software (GraphPad Software Inc., Boston, MA, USA) was used to generate graphs. The mean values and standard deviations (SD) were calculated from three replicates in this study.

## 5. Conclusions

In this study, the effects of salinity stress on adzuki bean seedlings were investigated through analyses of plant height, partial physicochemical properties of unifoliate leaves, and transcriptome profile. The results demonstrated that salinity stress could significantly inhibit plant height, induce O_2_^−^ accumulation, and increase enzymic activities of SOD, POD, and CAT, as well as pigments content at the low salt concentrations of the designed 32.5 mmol/L and 65.0 mmol/L NaCl levels. Transcriptome analysis suggested that adzuki bean seedlings adapted to salinity stress mainly through regulating expression patterns of genes involved in the spliceosome pathway, plant hormone signal transduction pathway, plant–pathogen interaction pathway, and MAPK signaling pathway. Our findings will help to improve the understanding of the mechanism of salt adaptation in adzuki bean seedlings, and bridge a critical gap in transcriptome study of this species under salt-treated conditions.

## Figures and Tables

**Table 1:** Summary of reads filtering and clean reads mapping in this study.

NaCl Concentration (mmol/L)	Sample	Total Raw Reads (M)	Total Clean Reads (M)	Total Clean Bases (Gb)	Clean Reads Q20 (%)	Clean Reads Q30 (%)	Clean Reads Ratio (%)	Total Mapping (%)	Unique Mapping (%)
0.0	CK1	43.82	42.3	6.35	97.75	93.78	96.53	86.91	78.30
	CK2	43.82	42.47	6.37	97.91	94.19	96.91	86.27	75.96
	CK3	43.82	42.7	6.41	97.62	93.41	97.45	86.01	77.86
32.5	T1_1	43.82	42.56	6.38	97.75	93.81	97.11	88.10	77.50
	T1_2	43.82	42.38	6.36	97.78	93.83	96.72	84.88	76.68
	T1_3	45.57	42.66	6.40	97.98	94.37	93.62	82.51	74.07
65.0	T2_1	43.82	42.5	6.38	97.82	93.97	96.99	87.13	77.97
	T2_2	43.82	42.15	6.32	97.91	94.20	96.18	86.04	76.52
	T2_3	43.82	42.56	6.38	97.75	93.75	97.13	85.80	77.15
Total		396.13	382.28	-	-	-	-	-	-

## Data Availability

The original contributions presented in this study are included in the article/Supplementary Material; further inquiries can be directed to the corresponding authors.

## Supplementary Materials

The following supporting information can be downloaded at https://www.mdpi.com/article/10.3390/plants14172722/s1, Figure S1: Ca/Cb in the unifoliate leaf of JXD6 seedlings treated with salinity stress. Different lowercase letters indicated statistical differences (*p* < 0.05) as evaluated by Duncan’s multiple range test method; Figure S2: Differentially expressed genes (DEGs) involved in abscisic acid (A), ethylene (B), and brassinosteroid (C) signaling transduction in salinity-treated adzuki bean seedlings. PYR/PYL: *ABA receptor PYR/PYL*; PP2C: *protein phosphatase 2C*; ERF1/2: *ethylene-responsive transcription factor 1/2*; SIMKK: *mitogen-activated protein kinase kinase 4/5*; BAK1: *brassinosteroid insensitive 1-associated receptor kinase 1*; BRI1: *protein brassinosteroid insensitive 1*; BSK: *BR-signaling kinase*; TCH4: *xyloglucan:xyloglucosyl transferase TCH4*; Table S1: Spliceosome related differentially expressed genes (DEGs); Table S2: Auxin signal transduction related differentially expressed genes (DEGs); Table S3: Cytokinin signal transduction related differentially expressed genes (DEGs); Table S4: Gibberellin signal transduction related differentially expressed genes (DEGs); Table S5: Salicylic acid signal transduction related differentially expressed genes (DEGs); Table S6: Abscisic acid, ethylene and brassinosteroid signal transduction related differentially expressed genes (DEGs); Table S7: Plant–pathogen interaction related differentially expressed genes (DEGs); Table S8: MAPK signaling pathways related differentially expressed genes (DEGs); Table S9: Alternative splice changes during salinity stress; Table S10: The information of primers used for RT-qPCR in this study.
