# Supplementary figures and images for: Transcriptome Analysis and Physiological Response to Salinity Stress in Adzuki Bean (Vigna angularis) at the Seedling Stage
Source: Plants (Basel). 2025 Sep 1;14(17):2722. doi: 10.3390/plants14172722 (PMC12430418; doi:10.3390/plants14172722)

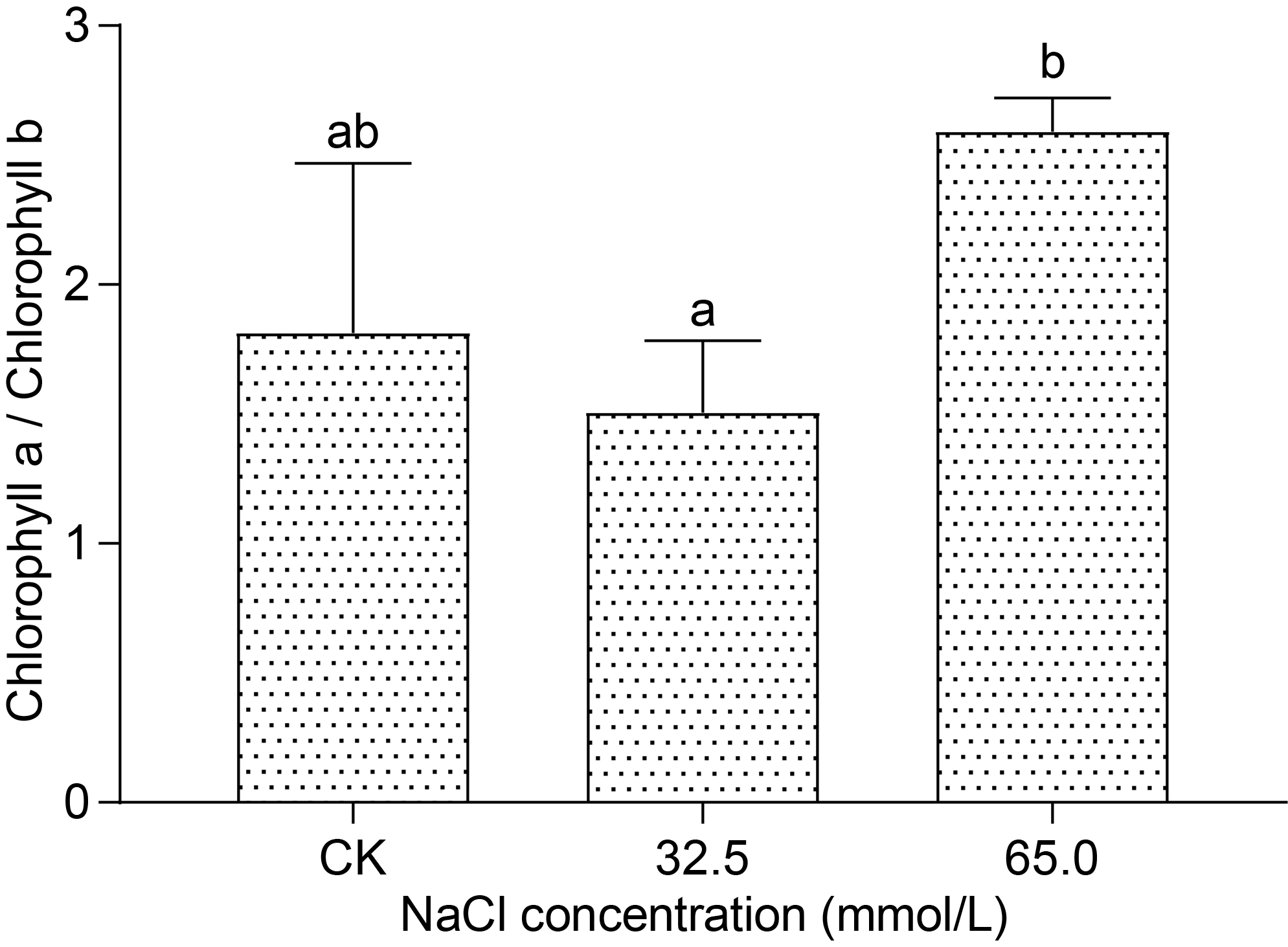

Supplement: Supplementary file 1 [file plants-14-02722-s001.zip › plants-3773541-supplementary/Supplementary materials/Figure S1.tif]

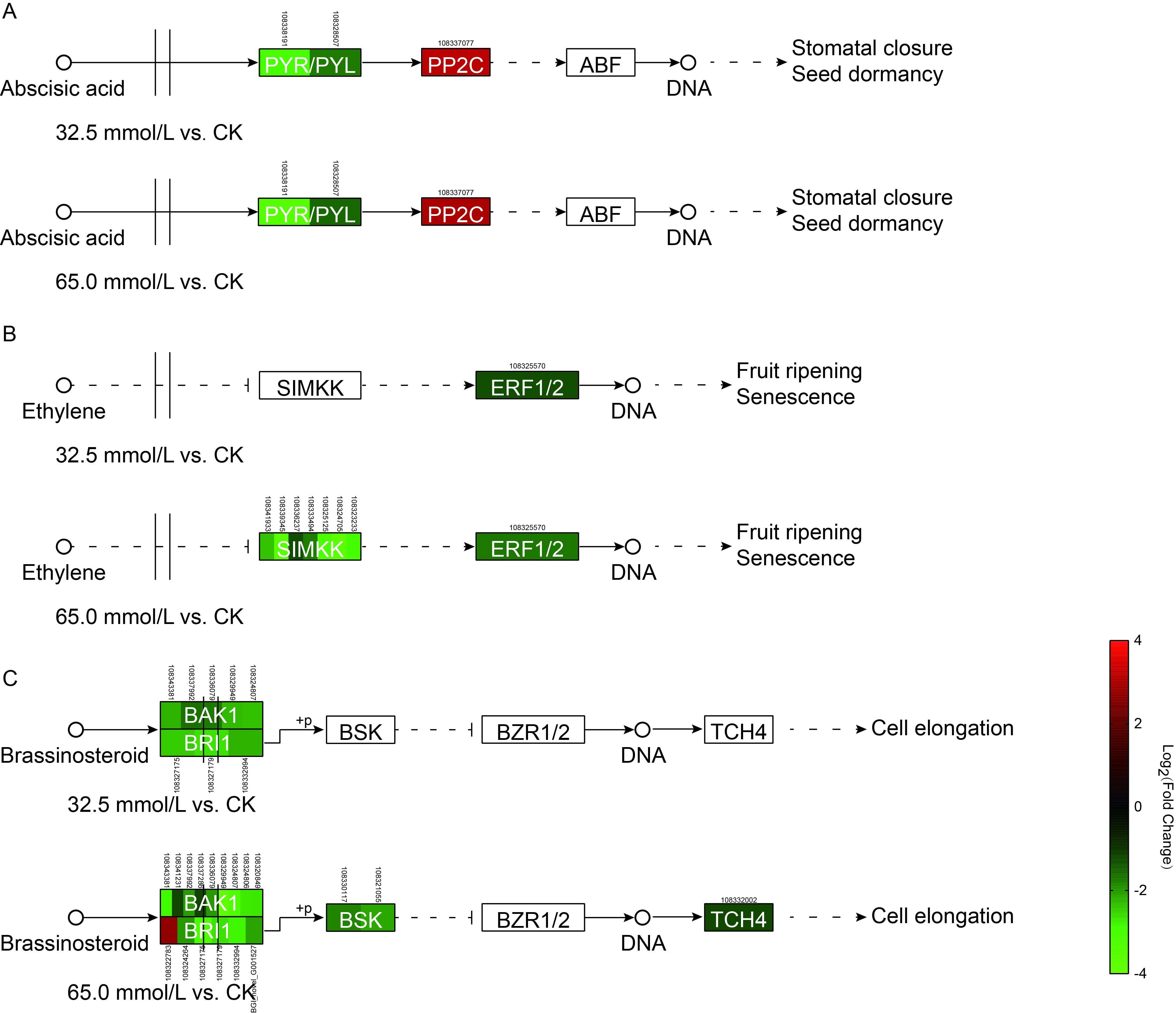

Supplement: Supplementary file 1 [file plants-14-02722-s001.zip › plants-3773541-supplementary/Supplementary materials/Figure S2.tif]
